# Evaluation of Probe Positioning Effects on Optical Parameters in Neonatal Forehead Time-Resolved Spectroscopy Measurements

**DOI:** 10.3390/bios16020069

**Published:** 2026-01-23

**Authors:** Yoko Tadatomo, Kota Inoue, Tomohito Nakayama, Aya Morimoto, Hiroaki Suzuki, Toru Kuboi, Kosuke Koyano, Shinji Nakamura, Takashi Kusaka

**Affiliations:** 1 Department of Pediatrics, Faculty of Medicine, Kagawa University, Takamatsu 760-0016, Kagawa, Japan; s23d716@kagawa-u.ac.jp (Y.T.); inoue.kota@kagawa-u.ac.jp (K.I.); kusaka.takashi@kagawa-u.ac.jp (T.K.); 2 Department of Neonatology, NHO Shikoku Medical Center for Children and Adults, Zentsuji 765-8507, Kagawa, Japan; kuboi.toru.am@mail.hosp.go.jp; 3 Medical Engineering Equipment Management Center, Kagawa University Hospital, Miki 761-0793, Kagawa, Japan; nakayama.tomohito@kagawa-u.ac.jp; 4 Maternal Perinatal Center, Faculty of Medicine, Kagawa University, Takamatsu 760-0016, Kagawa, Japan; morimoto.aya.1x@kagawa-u.ac.jp (A.M.); koyano.kosuke@kagawa-u.ac.jp (K.K.); 5 Central Research Laboratory, Hamamatsu Photonics K.K., Hamamatsu 430-8587, Shizuoka, Japan; hiro-su@crl.hpk.co.jp

**Keywords:** neonate, time-resolved spectroscopy, probe positioning

## Abstract

Time-resolved spectroscopy (TRS) is a promising tool for noninvasive cerebral monitoring in neonates. However, the optimal forehead site for probe placement remains unclear. In this study, we evaluated the effect of probe positioning on TRS-derived optical parameters in neonates. TRS measurements were obtained from the midline and right lateral forehead of 30 neonates (≥36 weeks’ corrected gestational age). We compared various parameters between the two probe positions, including optical intensity, attenuation, mean optical path length, scattering coefficient, total hemoglobin (tHb), cerebral oxygen saturation (ScO_2_) and cerebral blood volume (CBV). No significant differences were observed in tHb, ScO_2_ and CBV between the midline and lateral sites. However, the lateral site showed a significantly lower scattering coefficient and shorter mean path length. Light intensity was increased and attenuation was reduced at the lateral site. Thus, while tHb, ScO_2_ and CBV values were consistent between sites, the midline provided more stable scattering and optical path data. These findings suggest that the midline forehead may be a more suitable site for TRS-based neonatal cerebral monitoring.

## 1. Introduction

Near-infrared spectroscopy (NIRS) has become an increasingly important tool for noninvasive cerebral monitoring in neonatal intensive care units, enabling continuous assessment of cerebral oxygenation at the bedside. In recent years, large-scale, multicenter and multinational clinical studies have demonstrated the clinical relevance of cerebral oximetry in neonatal populations, particularly during critical periods such as the immediate transition after birth and the management of extremely preterm infants. A randomized phase 3 clinical trial (COSGOD III) showed that cerebral regional tissue oxygen saturation–guided oxygen delivery influenced early postnatal management in preterm neonates, highlighting the translational impact of cerebral NIRS monitoring in clinical practice [1]. Similarly, a large international cohort study reported the widespread use and clinical relevance of cerebral oximetry monitoring in extremely preterm infants across multiple countries [2]. These studies underscore the growing international acceptance of cerebral NIRS as a clinically meaningful monitoring modality in neonatology.

Conventionally, NIRS probes are placed on the lateral frontal region of the neonatal head to avoid optical interference from the superior sagittal sinus (SSS) [3], which lies beneath the anterior fontanelle in the midline [4,5]. This practice has been widely adopted to ensure stable measurements with minimal contamination from venous blood in large cerebral vessels [6]. Beyond cerebral hemodynamic monitoring, recent advances in optical technologies—including novel fiber-based approaches and image reconstruction techniques—are expanding the potential for optical sensing and recording of neural activity. These developments further underscore the importance of robust optical measurement strategies that can reliably account for tissue and geometric variability.

In recent years, quantitative near-infrared spectroscopy techniques have gained increasing attention in neonatal research, as they enable the absolute assessment of cerebral hemodynamic parameters rather than relative changes alone. Among these techniques, time-resolved spectroscopy (TRS) is distinguished by its ability to separate absorption and scattering properties through time-of-flight analysis of photons, allowing more robust estimation of total hemoglobin concentration (tHb), cerebral blood volume (CBV), and cerebral Hb oxygen saturation (ScO_2_) [7,8,9]. This feature is particularly advantageous in neonates, whose head anatomy, tissue composition, and vascular distribution differ substantially from those of older children and adults.

Despite these advantages, several technical and anatomical factors may influence TRS-derived parameters in clinical practice. Probe positioning on the neonatal forehead is one such factor that has been largely guided by convention rather than direct experimental validation. Lateral frontal placement has been widely recommended to avoid potential contamination from the superior sagittal sinus beneath the anterior fontanelle [10]. However, this recommendation has primarily been derived from studies using continuous-wave NIRS and theoretical considerations, and it remains unclear whether it is equally applicable to TRS, which emphasizes tissuephoton trajectories and absolute quantification.

Moreover, the neonatal forehead is not a uniform optical surface. Differences in curvature, scalp–skull geometry, and underlying brain structure between the midline and lateral regions may affect photon propagation, optical path length, and scattering characteristics. Previous TRS studies in neonates have demonstrated that optical properties and hemodynamic parameters vary with developmental stage and measurement conditions [11]. However, it remains unknown whether such site-dependent optical differences translate into differences in clinically relevant indices when measured using TRS.

Therefore, clarifying the impact of probe positioning on both optical properties and hemodynamic parameters is essential for optimizing TRS-based neonatal cerebral monitoring and for establishing evidence-based guidelines for probe placement in both clinical and research settings.

The aim of this study was to evaluate whether NIRS-derived parameters—specifically, tHb, CBV, ScO_2_ and optical properties—differ significantly between midline and lateral frontal probe positions in neonates. Using TRS, which provides absolute values of hemoglobin concentration and optical scattering, we conducted a clinical investigation to determine whether conventional probe placement recommendations are justified.

## 2. Materials and Methods

This prospective observational study was conducted at Kagawa University Hospital. The protocol was approved by the Regional Committee on Biomedical Research Ethics of Kagawa University (approval number: H29-042), and written informed consent was obtained from the parents of all neonates prior to participation. The study was performed in accordance with the Declaration of Helsinki.

We enrolled 30 neonates with a corrected gestational age of ≥36 weeks who were admitted to the neonatal intensive care unit and growing care unit in our hospital from January to December 2024. Exclusion criteria were as follows: (1) requirement for any respiratory support, such as supplemental oxygen, continuous positive airway pressure or mechanical ventilation; (2) congenital anomalies, including heart disease; and (3) abnormal optical properties as defined previously [12]. Demographic information, including gestational age, birth weight, Apgar scores and head circumference, was collected from medical records.

**Figure 1:**
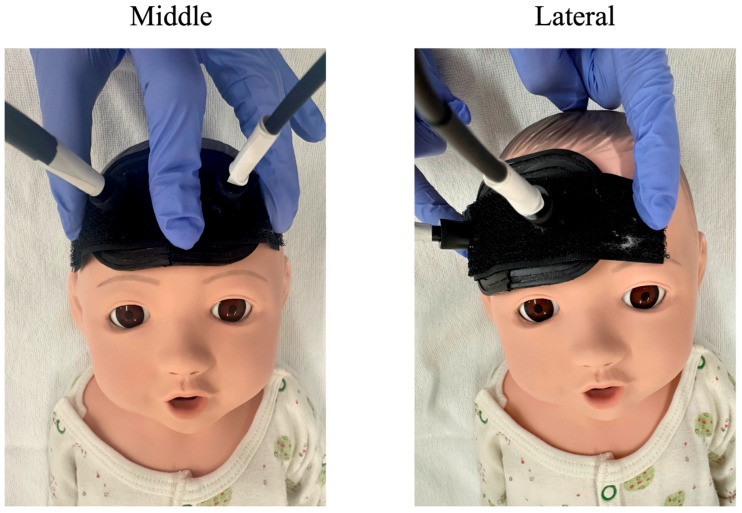
Schematic representation of time-resolved spectroscopy probe placement on the neonatal forehead. The optical probes were placed either on the midline or on the right lateral forehead of the neonate, demonstrated here using a neonatal manikin.

Measurements were performed using a portable three-wavelength near-infrared TRS system (TRS-21, Hamamatsu Photonics K.K., Hamamatsu, Japan). This device employs a time-correlated single-photon counting technique with light pulses of 762, 801 and 836 nm. The source–detector distance was fixed at 30 mm using a probe. Probes were sequentially placed on the midline and right lateral forehead of each neonate (Figure 1). Each measurement was performed for 5 min. Measurements were conducted in stable neonates placed in a cot during sleep or quiet rest. To ensure stable and reproducible measurements, all TRS recordings were performed under standardized conditions. Measurements were conducted while the neonates were in a calm state, either asleep or at quiet rest, and no handling or clinical interventions were performed during data acquisition. Heart rate and peripheral oxygen saturation (SpO_2_) were continuously monitored throughout data acquisition. The mean heart rate during measurements was approximately 130 beats per minute, and the mean SpO_2_ was 98%, with no clinically relevant fluctuations observed. The probe was gently fixed to the skin using adhesive tape, and care was taken to maintain consistent probe–skin contact throughout the measurement period.

For each probe position, data were continuously acquired for 5 min, and segments with visible motion artifacts or unstable photon count rates were excluded from analysis. Optical signals were monitored in real time to confirm sufficient photon counts and stable temporal profiles. Only datasets that met predefined quality criteria, including stable count rates and consistent temporal dispersion profiles, were included in the final analysis.

To minimize inter-measurement variability, the same TRS device and probe configuration were used for all measurements, and all recordings were performed by trained personnel following an identical measurement protocol. Sequential measurements at the midline and lateral forehead were completed within a short time interval in each neonate to reduce the influence of physiological fluctuations. These procedures were implemented to enhance measurement stability and reproducibility across subjects and probe positions.

The re-emission profiles were analyzed using the photon diffusion equation described by Patterson et al., convoluted with the instrumental response function, to calculate the absorption coefficient (μ_a_) and reduced scattering coefficient (μ_s_′) at three wavelengths. From these coefficients, oxyhemoglobin (oxyHb) and deoxyhemoglobin (deoxyHb) concentrations were determined, and tHb, ScO_2_ and CBV were calculated as follows:

(1) $${µa}_{762nm}=\varepsilon_{762nm}^{oxyHb}\left[oxyHb\right]+\varepsilon_{762nm}^{deoxyHb}\left[deoxyHb\right]+{µa}_{762nm}^{background}$$

(2) $${µa}_{801nm}=\varepsilon_{801nm}^{oxyHb}\left[oxyHb\right]+\varepsilon_{801nm}^{deoxyHb}\left[deoxyHb\right]+{µa}_{801nm}^{background}$$

(3) $${µa}_{836nm}=\varepsilon_{836nm}^{oxyHb}\left[oxyHb\right]+\varepsilon_{836nm}^{deoxyHb}\left[deoxyHb\right]+{µa}_{836nm}^{background}$$

In these equations, $\varepsilon_{\lambda \mathrm{nm}}^{x}$ is the extinction coefficient at λ nm, and [oxyHb] and [deoxyHb] are the concentrations of oxyHb and deoxyHb, respectively.

(4) [totalHb]=[oxyHb]+[deoxyHb]

(5) $$CBV(ml/100g\;brain\;tissue)={\left[totalHb\right]\times MW}_{Hb}\times 10^{-6}/(tHb\times 10^{-2}\times Dt\times 10)$$

(6) $${ScO}_{2}(\%)=\{[oxyHb]/([oxyHb]+[deoxyHb])\}\times 100$$

where [ ] indicates the Hb concentration (μM), MW_Hb_ is the molecular weight of Hb (64,500), tHb is the venous Hb concentration (g/dL) and Dt is the brain tissue density (1.05 g/mL).

Optical parameters (light intensity, attenuation, mean optical path length and μ_s_′), as well as tHb, ScO_2_ and CBV, were compared between the midline and lateral forehead sites. Paired *t*-tests or Wilcoxon signed-rank tests were used, as appropriate. A *p*-value of <0.05 was considered statistically significant. Statistical analyses were performed using GraphPad Prism version 10.6.0 (796) (GraphPad Software, San Diego, CA, USA).

## 3. Results

### 3.1. Participants and Measurement Timing

The gestational age at birth ranged from 28 + 1 weeks to 40 + 5 weeks, with a median of 36 + 0 weeks. The median birth weight was 2121 g. The age at measurement ranged from 1 to 85 days, with a median of 16 days. The corrected gestational age at the time of TRS measurement ranged from 36 + 2 weeks to 42 + 6 weeks, with a median of 38 + 1 weeks. Head circumference ranged from 30.5 to 35.5 cm, with a median of 33.05 cm. The sex distribution was 15 males and 15 females.

### 3.2. Optical Intensity and Attenuation Level

**Figure 2:**
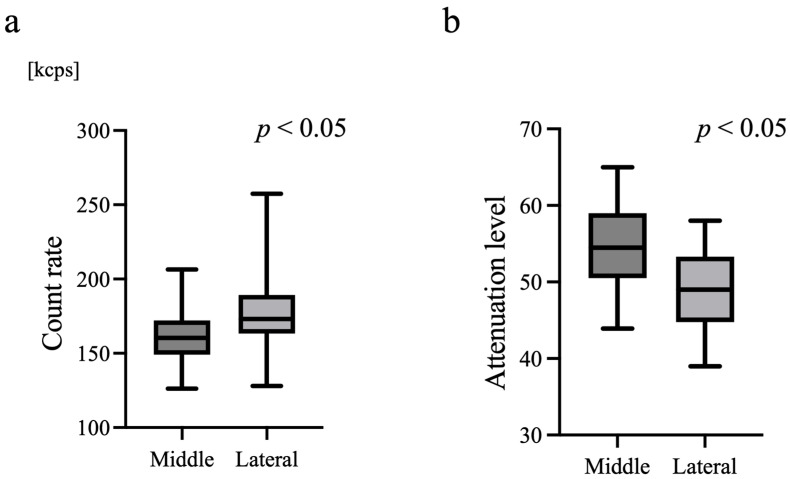
Comparison of light intensity and attenuation between midline and lateral forehead sites. (**a**) Count rate of detected photons and (**b**) the attenuation level measured at the midline and right lateral forehead. Count rate was significantly higher and attenuation level was significantly lower at the lateral site compared to the midline (*p* < 0.05).

The light intensity (count rate) (Figure 2a) was significantly higher at the right lateral forehead, whereas the attenuation level (Figure 2b) was significantly higher at the midline (*p* < 0.05). These differences were consistently observed across all measured wavelengths.

### 3.3. Mean Optical Path Length and Scattering Coefficient

**Figure 3:**
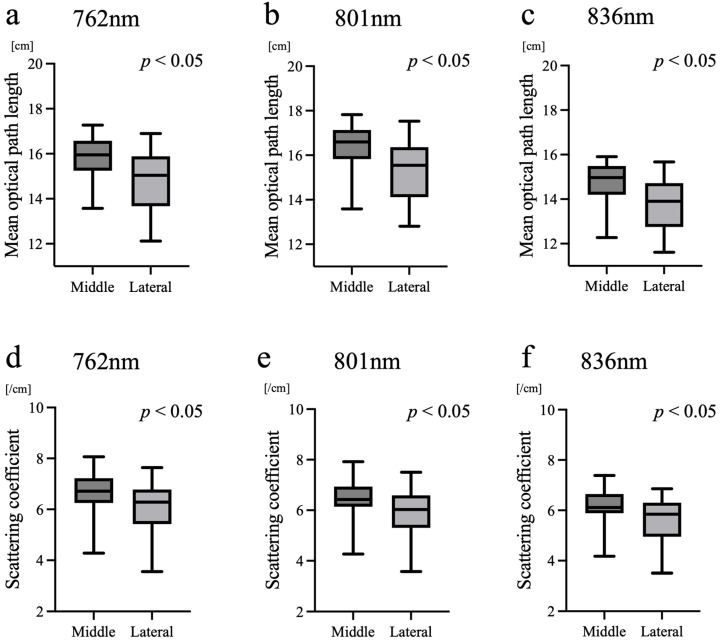
Comparison of the mean optical path length and reduced scattering coefficient. (**a**–**c**) Mean optical path length and (**d**–**f**) reduced scattering coefficient (μ_s_′) obtained from TRS measurements at the midline and right lateral forehead. Both indices were significantly smaller at the lateral site than at the midline (*p* < 0.05).

The mean optical path length was significantly shorter (Figure 3a–c) and the reduced scattering coefficient (Figure 3d–f) was significantly smaller at the lateral site than at the midline (*p* < 0.05). The shorter mean optical path length and lower reduced scattering coefficient at the lateral site were observed at all three wavelengths and in the majority of subjects.

### 3.4. Absorption Coefficient, tHb, ScO_2_ and CBV

**Figure 4:**
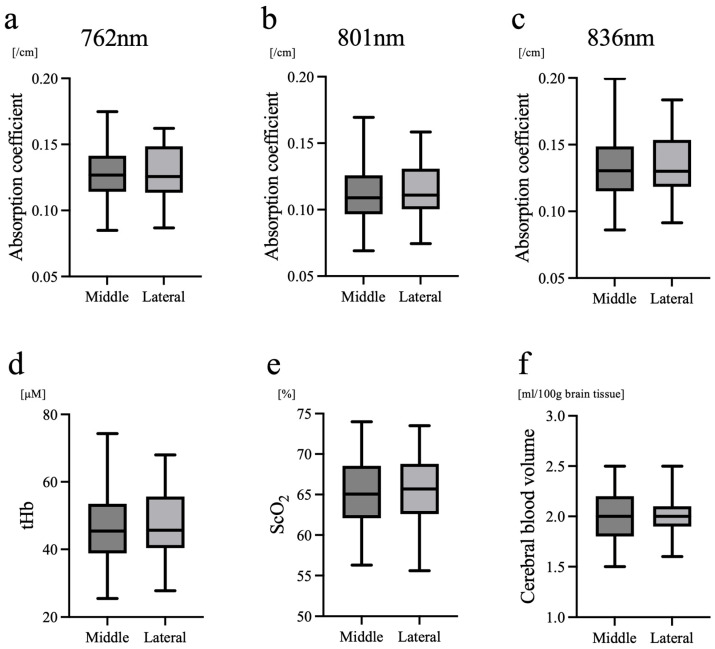
Absorption coefficient, total hemoglobin concentration, cerebral oxygen saturation and cerebral blood volume. Comparison of (**a**–**c**) absorption coefficient (μ_a_), (**d**) total hemoglobin concentration (tHb), (**e**) cerebral oxygen saturation (ScO_2_) and (**f**) cerebral blood volume between the midline and right lateral forehead sites. No significant differences were observed between the two positions.

There were no significant differences between the midline and lateral sites in terms of the absorption coefficient (Figure 4a–c), tHb (Figure 4d), and ScO_2_ and CBV (Figure 4e,f). No significant differences in the absorption coefficient, tHb, ScO_2_, or CBV were observed between the midline and lateral sites across all measured wavelengths.

## 4. Discussion

The principal findings of this evaluation of the impact of probe position on NIRS-derived parameters were as follows: (1) there were no significant differences in tHb, ScO_2_ and CBV between the midline and lateral forehead positions, and (2) light intensity was higher and attenuation level was lower at the lateral site, while mean optical path length and the reduced scattering coefficient (μ_s_′) were also significantly smaller. These results indicate that oxygenation indices obtained with TRS (tHb, ScO_2_ and CBV) are robust against probe positioning, whereas optical parameters related to signal quality may be influenced by site.

The absence of differences in tHb, ScO_2_ and CBV is consistent with the principle of TRS, which emphasizes signal contributions from cerebral tissue by using time-resolved photon information. Although lateral placement has historically been recommended to avoid the influence of the SSS beneath the anterior fontanelle [3], our data showed no differences in absorption, tHb, ScO_2_ and CBV according to site. This suggests that TRS, through its depth sensitivity and ability to provide absolute quantification, effectively suppresses contamination from large superficial vessels even near the midline in neonates.

In contrast, the uniformly higher value of light intensity and lower values of attenuation, mean optical path length and scattering at the lateral site (Figure 2 and Figure 3) are most likely attributable to geometric and coupling differences. The midline forehead is relatively flat, allowing stable probe–skin contact, whereas the lateral forehead is more curved, which may shorten the effective source–detector distance and alter photon scattering angles. These factors shorten the estimated optical path length, leading to smaller scattering estimates, consistent with our findings.

In contrast to previous reports that primarily compared larger anatomical regions, such as the frontal and temporal areas [13], the present study focused on a subtle positional difference between the midline and lateral forehead. While regional structural factors of the brain, including differences in white matter volume and the depth of the subarachnoid space, may partly explain the observed variation in μ_s_′ [11,14], it is also likely that technical aspects related to probe–skin contact and the local geometry of the attachment site substantially contributed to the results.

An additional consideration is the physiological interpretation of the present findings. Although the scattering coefficient and mean optical path length differed significantly between the midline and lateral regions, the hemoglobin concentration and ScO_2_ remained consistent. This paradox suggests that TRS-derived hemodynamic parameters are not merely influenced by local optical geometry but primarily reflect the vascular and metabolic status within the cerebral tissue. Several mechanisms may account for the discrepancy between the site-dependent differences in scattering and path length and the absence of differences in tHb and ScO_2_. First, the mean optical path length was consistently shorter at the lateral forehead, likely because the probe sits on a more curved surface. This results in a shallower fiber–skin angle and a reduced effective photon entry angle. This geometric effect limits photon penetration and leads to a shorter estimated path length at the lateral site. Second, Monte Carlo simulations of neonatal head models indicate that deeper photon trajectories increase the relative contribution of white matter to the detected signal. Because neonatal white matter exhibits approximately twice the reduced scattering coefficient of gray matter (μ_s_′ = 1.0/mm vs. 0.5/mm), deeper penetration at the midline would be expected to yield higher overall scattering values, consistent with our findings [15]. Third, absorption properties are known to differ far less between gray and white matter than scattering properties, according to a previous optical study. Moreover, by the gestational ages at which our measurements were performed, the vascular densities of the cortex, subcortical white matter, and deep white matter have been reported to become relatively similar. Therefore, the increased sampling depth at the midline may not substantially alter the volume-averaged absorption coefficient [16]. This could explain why tHb did not differ between the two sites, despite clear site-dependent differences in scattering and optical path length. This interpretation is consistent with previous TRS studies in neonates, showing that developmental and anatomical factors influence optical properties such as scattering and path length, whereas hemodynamic indices derived from absorption are relatively preserved across measurement conditions, supporting the robustness of TRS-based cerebral oxygenation assessment [11]. The comparable tHb and ScO_2_ values observed despite site-dependent differences in scattering and optical path length highlight an important methodological advantage of TRS. By independently estimating absorption and scattering properties, TRS-derived hemodynamic parameters appear less sensitive to geometric variations. In contrast, simplified approaches that assume fixed scattering values may be more susceptible to site-dependent measurement errors when probe geometry or tissue composition differs.

Finally, ScO_2_ reflects the balance of arterial and venous Hb oxygenation and their relative volume fractions, which do not appear to differ systematically between the two forehead sites, explaining the comparable ScO_2_ values. In other words, even though the optical scattering characteristics vary according to structural differences, the hemodynamic information obtained by TRS appears to be independent of the tissue microstructure. This may imply that TRS has the potential to distinguish between structural and circulatory properties, measuring them separately rather than as a composite signal. While the method assumes optical homogeneity within the sampled volume, our results indicate that such an assumption does not necessarily compromise hemodynamic estimation, provided that measurements are performed at a fixed site under consistent conditions.

Beyond technical stability, our findings have physiological implications. The frontal midline anatomically corresponds to the watershed area between the anterior and middle cerebral arteries, which is particularly susceptible to hypoxic–ischemic injury in neonates. Reliable monitoring at this site, therefore, provides a physiologically meaningful window into global cerebral perfusion. Importantly, the SSS lies immediately beneath the scalp at the midline, and conventional continuous-wave NIRS is easily influenced by its venous signals. In contrast, TRS, by resolving the photon time-of-flight distribution, selectively captures photons that might penetrate tissue layers and thus minimizes the contribution of large superficial vessels such as the sagittal sinus. Consequently, midline TRS measurements are likely to reflect the oxygenation status of the underlying cortical and subcortical tissue rather than the venous blood in the sinus itself. This feature enhances the physiological relevance of midline monitoring, providing a noninvasive means to assess cerebral oxygenation in regions most vulnerable to injury.

There are some limitations in this study, First, this was a single-center study with a moderate sample size, leaving uncertainty in effect size estimates for some parameters. Second, only the right lateral forehead was tested, and potential left–right differences were not examined. Third, we did not quantify individual variations in scalp/skull thickness, forehead curvature or probe–scalp coupling, including contact pressure and potential hair interference, all of which may have contributed to geometric effects. Although probe placement was carefully performed by experienced operators with consistent manual fixation and attention to avoiding hair whenever possible, objective measures of probe–scalp coupling were not assessed. In addition, the TRS-21 probe is relatively bulky, and optimal adaptation to the curved neonatal scalp may differ between midline and lateral (temporal) positions. Therefore, some of the observed differences in optical parameters between midline and lateral measurements may reflect positioning-related technical factors rather than true anatomical tissue differences. Fourth, the distance to the SSS was not individually assessed by imaging, limiting our adjustment for anatomical diversity. Fifth, the cohort mainly consisted of neonates at 37–40 weeks’ corrected gestational age, so extrapolation to more immature infants should be approached with caution. Finally, the study did not assess stability or artifact resistance during prolonged continuous monitoring. In addition, midline and lateral measurements were performed sequentially rather than simultaneously. Although recordings were completed within a short time interval under clinically stable conditions, transient physiological variability during sequential acquisition cannot be entirely excluded and may have influenced the observed differences.

Future work should: (1) identify the position and depth of the SSS by ultrasound or MRI and examine its relationship with TRS indices; (2) evaluate reproducibility and motion tolerance through repeated measurements at multiple sites and time points, including both lateral sides; (3) assess how developmental changes in head circumference and cranial curvature affect site-related differences; (4) extend validation to preterm infants and hemodynamically unstable cases; and (5) apply Monte Carlo simulations to clarify the effects of curvature and optical properties. Such efforts will advance mechanistic understanding and practical optimization of probe placement for TRS in neonatal monitoring.

To conclude, in neonatal forehead TRS measurements, tHb, ScO_2_ and CBV values are comparable between midline and lateral sites, whereas scattering and optical path length are more stable at the midline. From the perspective of oxygenation monitoring, probe positioning may be flexible. However, for the reliable interpretation of optical parameters, midline placement is preferable. These findings contribute to the optimization of practical probe placement strategies in neonatal cerebral monitoring and highlight the need for further validation that considers anatomical diversity and pathological conditions. In clinical practice, trends in ScO_2_, tHb, and CBV are the most clinically relevant indices. The present results indicate that these parameters are comparable between midline and lateral forehead placements, supporting flexibility in probe positioning for routine cerebral oxygenation and circulatory monitoring. However, when stable estimation of optical properties is required, particularly in research-oriented applications or under low-signal conditions, midline placement may be preferable.

## Data Availability

The datasets generated during and/or analyzed during the current study are available from the corresponding author on reasonable request.

## Abbreviations

The following abbreviations are used in this manuscript:

CBV	Cerebral blood volume
deoxyHb	Deoxyhemoglobin
NIRS	Near-infrared spectroscopy
oxyHb	Oxyhemoglobin
ScO_2_	Cerebral Hb oxygen saturation
SSS	Superior sagittal sinus
tHb	Total hemoglobin concentration
TRS	Time-resolved spectroscopy
